# Supplementary material for: Isochronic development of cortical synapses in primates and mice
Source: Nat Commun. 2023 Dec 4;14:8018. doi: 10.1038/s41467-023-43088-3 (PMC10695974; doi:10.1038/s41467-023-43088-3)
Supplement: Supplementary file 1 — Supplementary Information [file 41467_2023_43088_MOESM1_ESM.pdf]

Supplementary Figures

Supplementary Figure 1

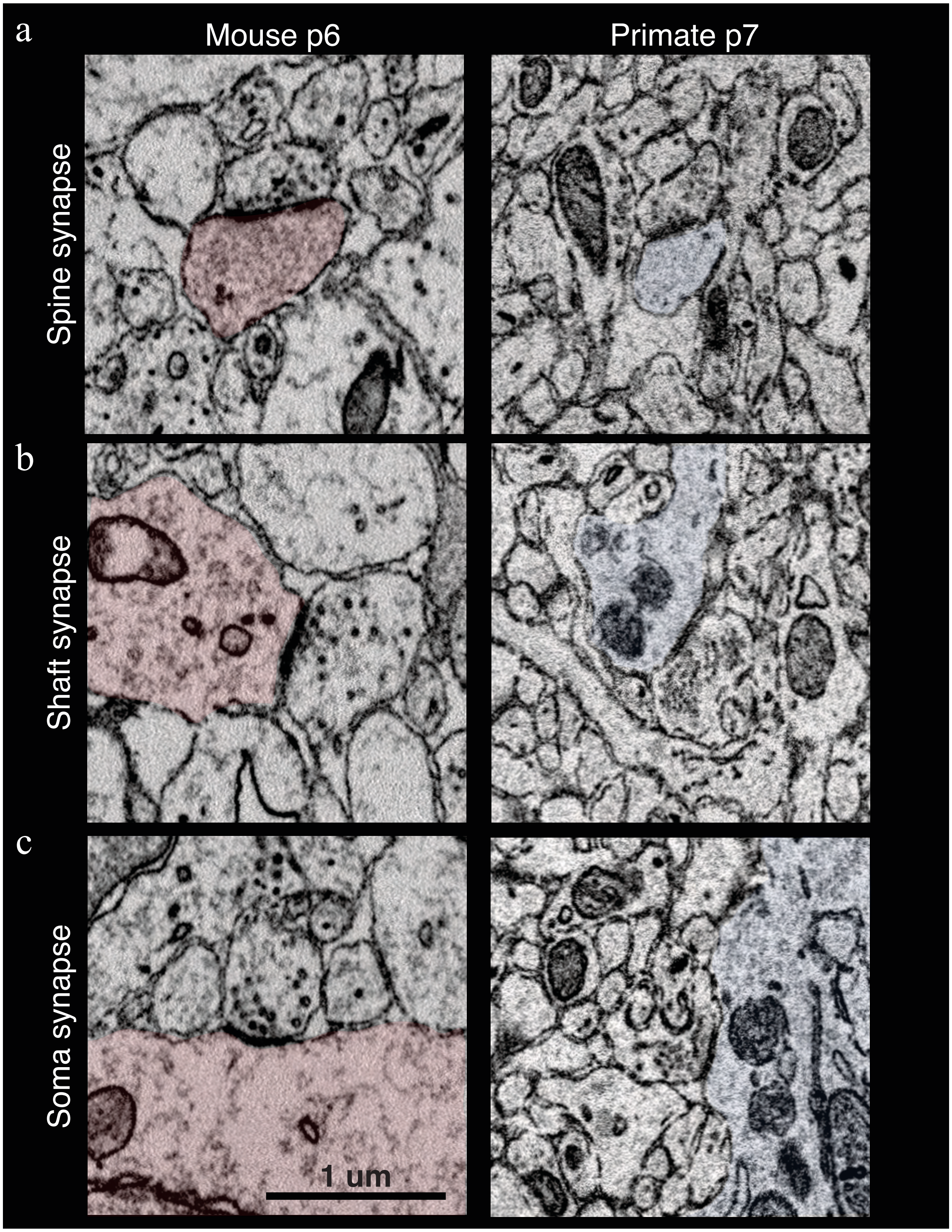

Supplementary Figure 1. Synapses of the p6 mouse and p7 primate. Representative 2D EM images of **(a)** excitatory spine, **(b)** inhibitory shaft, and **(c)** inhibitory soma synapses in p6 mouse (left) and p7 primate (right). In each image, the post-synaptic target is colored in red (mouse) or blue (primate). Note the prominent PSD and pre-synaptic vesicle cloud established early in neonatal development. Scale bar = 1 μm.

Supplementary Figure 2

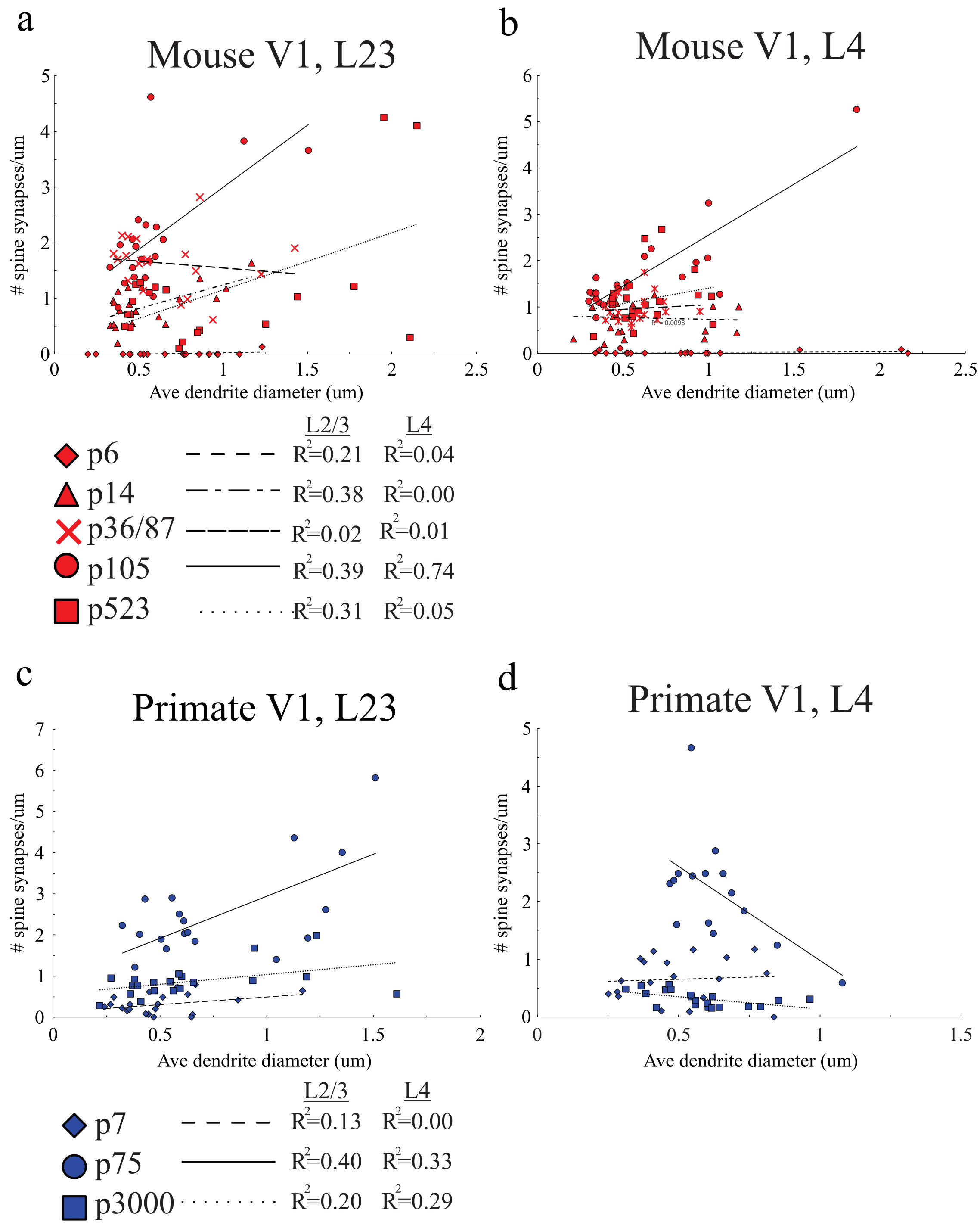

Supplementary Figure 2. Excitatory spine synapse density relationship to dendrite diameter. Scatter plot of x: average diameter of quantified 10 μm dendrite fragment and y: # of spine synapses/μm along the 10 μm dendrite fragment for mouse V1, L2/3 **(a)** and L4 **(b)**, and primate V1, L2/3 **(c)** and L4 **(d)**. Key for mouse (red) and primate (blue) show line-type representing best linear fit along with corresponding r^2 value for each data set. Linear regression used for r^2 calculation. Total synapse counts are identical to Figure 1b. Source data are provided as a Source Data file.

Supplementary Figure 3

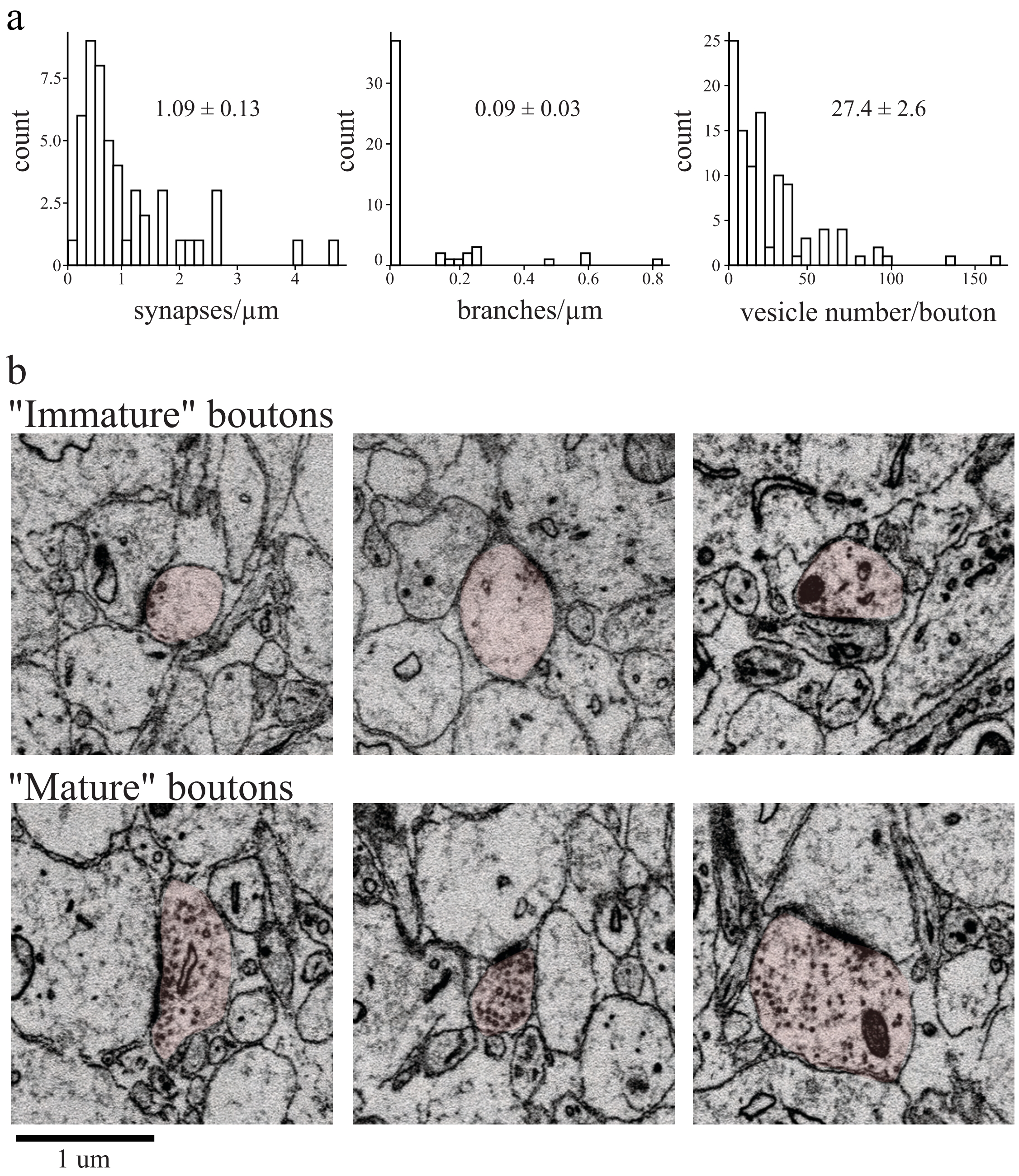

Supplementary Figure 3. Mouse p6 L2/3 axons make synapses but rarely branch and have fewer vesicles. **a,** Histograms of p6 axon synapses/µm *(left)*, branches/µm *(middle)*, and vesicle number/bouton *(right)*. **b**, Representative 2D EM images of mouse p6 L2/3 axons with *top*: ”immature” boutons that have a PSD but very few vesicles and *bottom*: ”mature” boutons with prominent PSD and large vesicle cloud. Boutons shaded in red for visualization purposes. Scale bar = 1 µm. mean±sem listed for each graph, n = 111 synapses across 50 axons. Source data are provided as a Source Data file.

Supplementary Figure 4

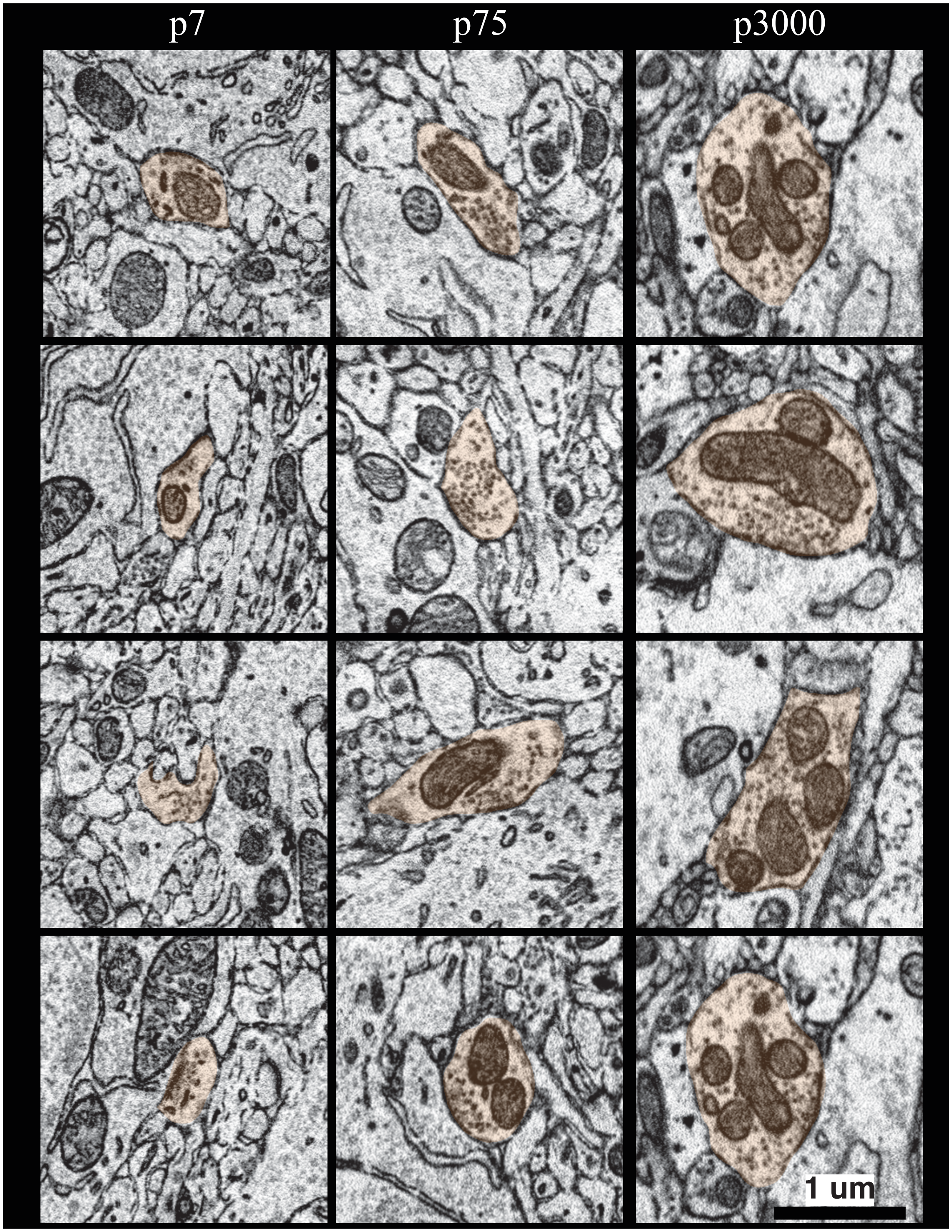

Supplementary Figure 4. Boutons of synapses onto primate excitatory neuron soma increase in size over development. 2D EM images showing a column of four representative axonal boutons (highlighted in orange) making a synapse onto primate excitatory soma at the noted ages. Scale bar = 1 μm.

Supplementary Figure 5

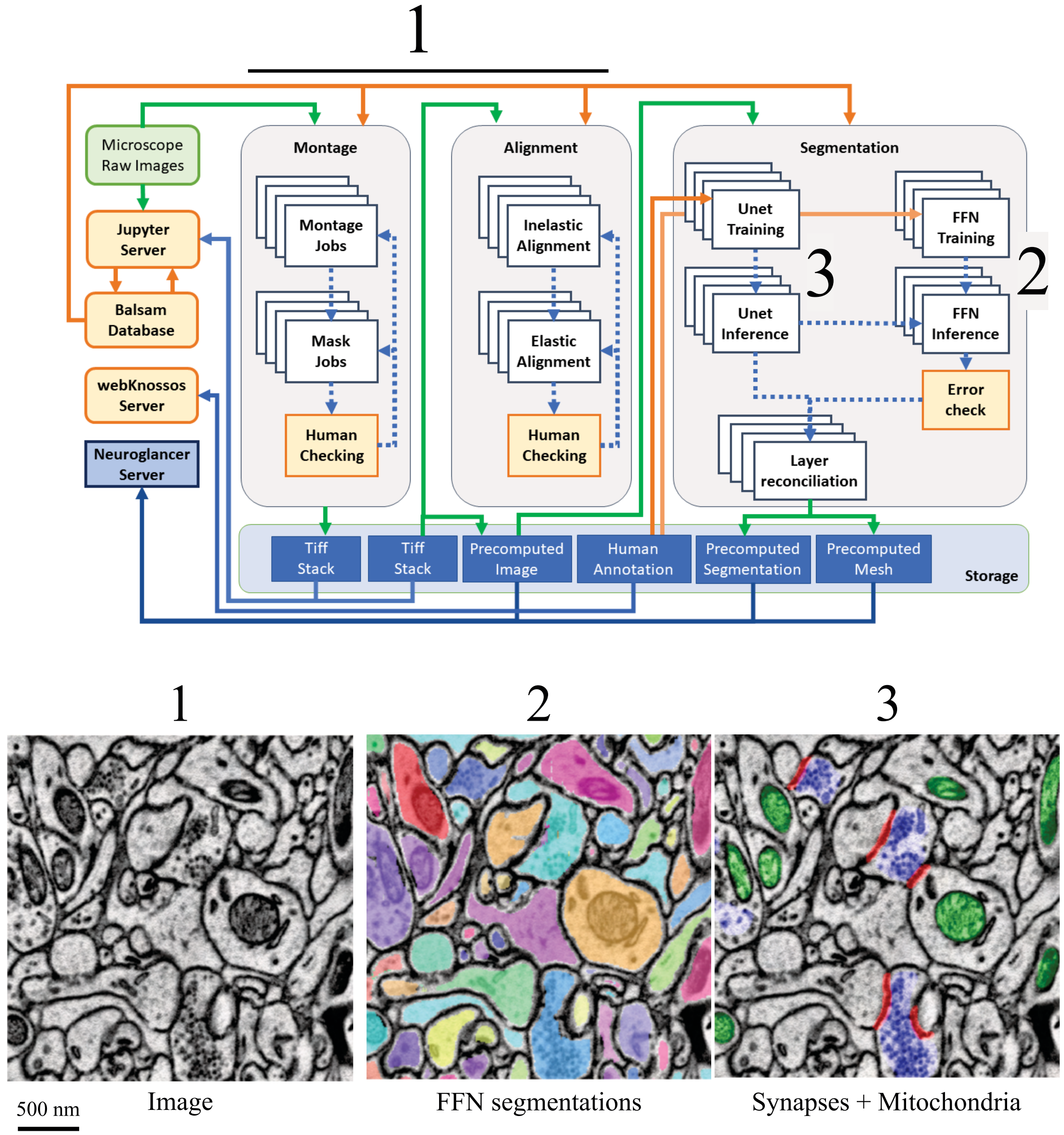

Supplementary Figure 5. Schematic of the automatic segmentation pipeline. *Left:* The green box represents the data acquisition. Orange boxes and arrows represent human interactions in the pipeline. The white boxes represent High Performance Computing (HPC)-submitted jobs. Blue boxes represent the data storage and visualization server. Green arrows represent I/O from the computing resources. *Bottom:* Representative images generated from different steps in the pipeline: 1 = raw images produced from 2D montage and 3D alignment, 2 = FFN saturated segmentation of all neuronal processes, and 3 = UNET/connected components sparse segmentation of synapses (red=PSD and blue=vesicle cloud) and mitochondria (green). Scale bar = 500nm.

Supplementary Figure 6

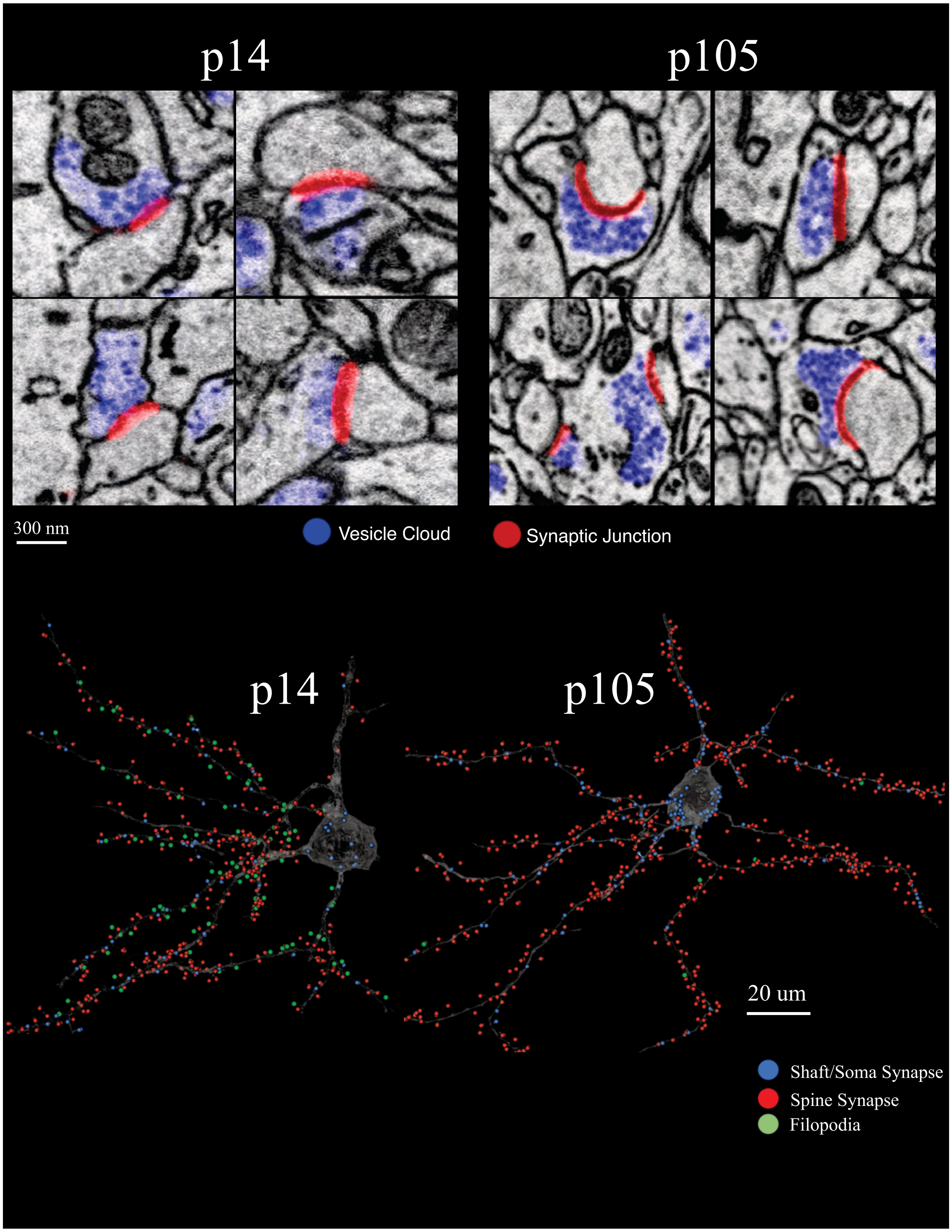

Supplementary Figure 6. Automatic segmentation of synapse vesicle cloud and PSD. *Top:* Representative 2D EM images showing automatically identified synapses in mouse p14 and p105 datasets. blue colored regions mark the pre-synaptic vesicle cloud, and red lines mark the PSD. *Bottom:* Representative rendering of a p14 (left) and p105 (right) neuron derived from FFN segmentation overlayed with UNET segmentation of shaft and soma synapses (blue circles), spine synapses (red circles), and filopodia (green circles).

Supplementary Figure 7

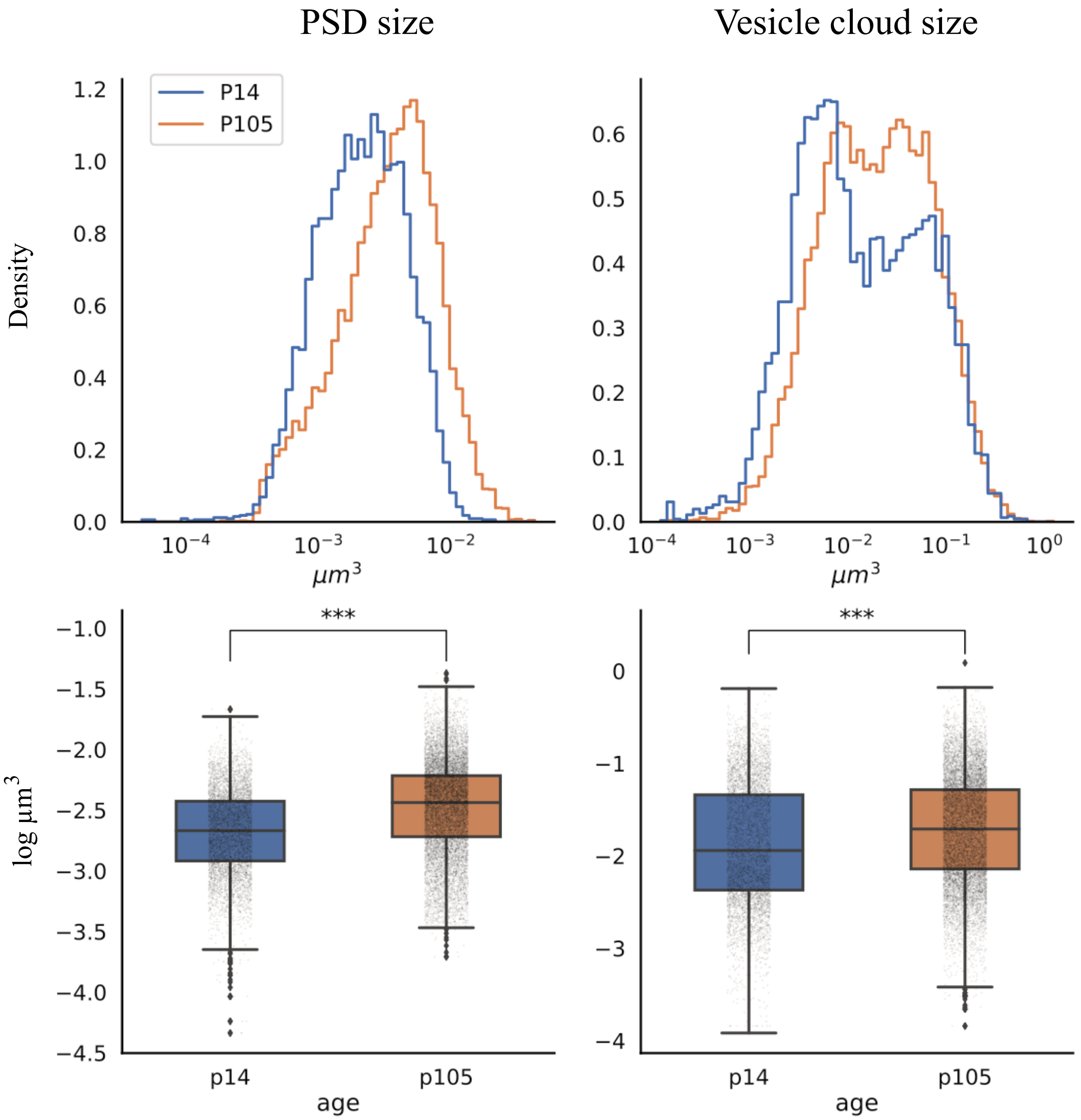

Supplementary Figure 7. Quantification of spine synapse changes from manually-error checked, automatic segmentation in mouse p14 and p105, L4. Changes in PSD size *(Left)*, and vesicle cloud size *(Right)* between mouse p14 (blue) and p105 (orange) V1,L4. Each analysis is represented as both a density (top) and box plot (bottom). For boxplots, the midline defines the mean, boxes show the interquartile range and lines define the min/max value. Two-tailed Mann-Whitney U test. $p < 0.0001$ for both results. Source data are provided as a Source Data file.

Supplementary Figure 8

a

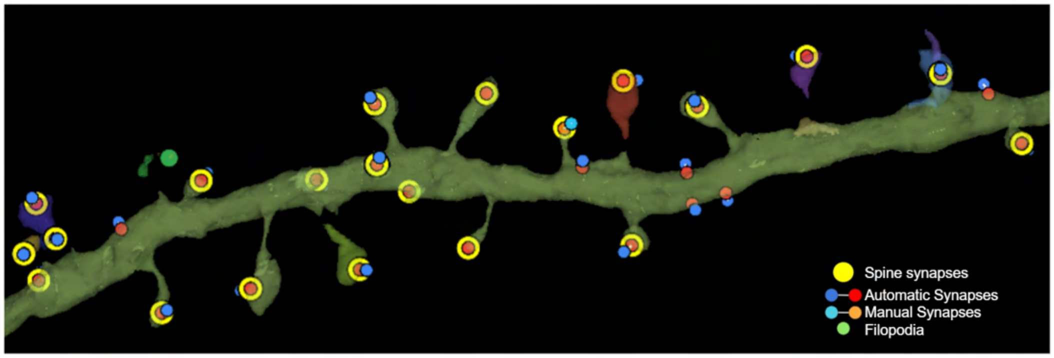

b

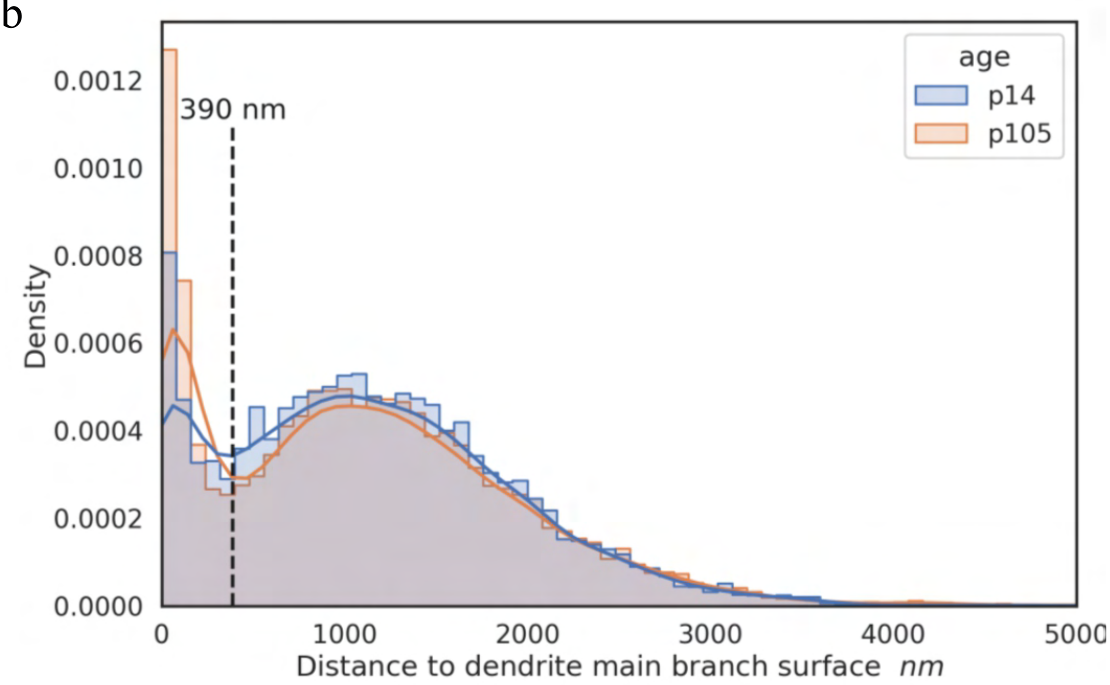

Supplementary Figure 8. Distance of spine synapse PSD from dendritic shaft in p14 and p105 mouse V1, L4. a, Representative reconstruction of an excitatory dendrite with spine synapses marked in yellow, filopodia in green, and stick-and-ball segments from automatic (dark blue-red) and manual (light blue-orange) annotations used for measuring the euclidian distance between the PSD and dendrite shaft. **b,** Histogram of distance from PSD to dendrite surface (i.e., shaft) for mouse V1, L4 excitatory neurons in at ages p14 (blue) and p105 (orange). A 390 nm cut-off was used to differentiate shaft (<390nm) from spine (>390nm) synapses. Two-tailed Mann-Whitney U test. $p = 0.80$. Total automatically segmented and manually proofread synapse counts for b: mouse p14 = 6809, p105 = 13509. Source data are provided as a Source Data file.

Supplementary Figure 9

a

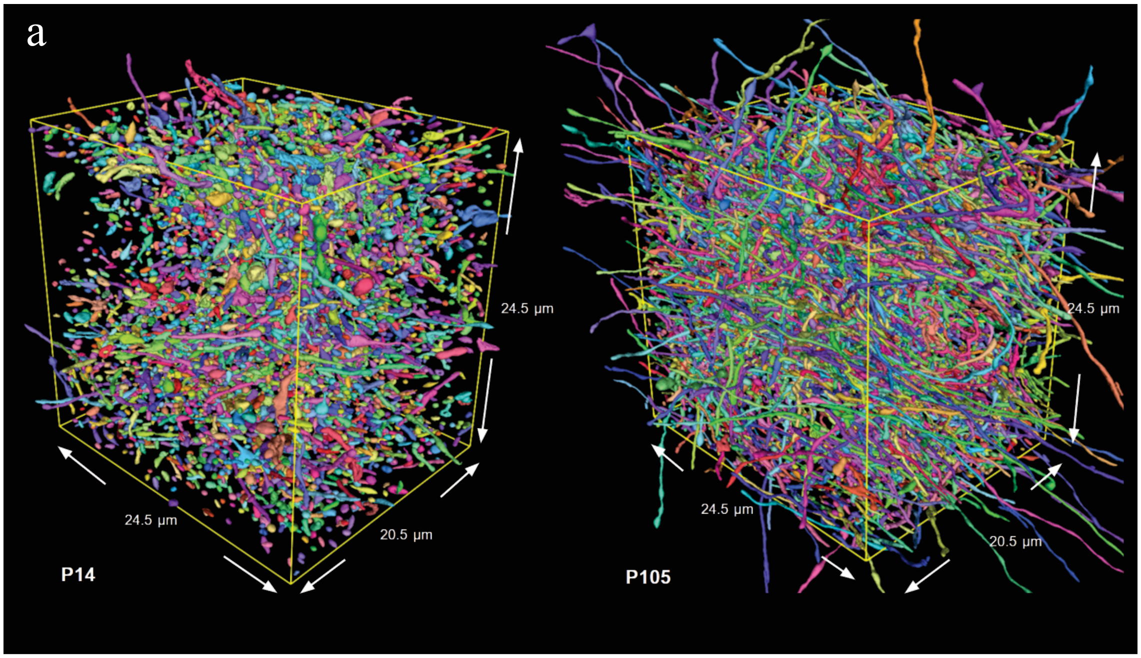

b

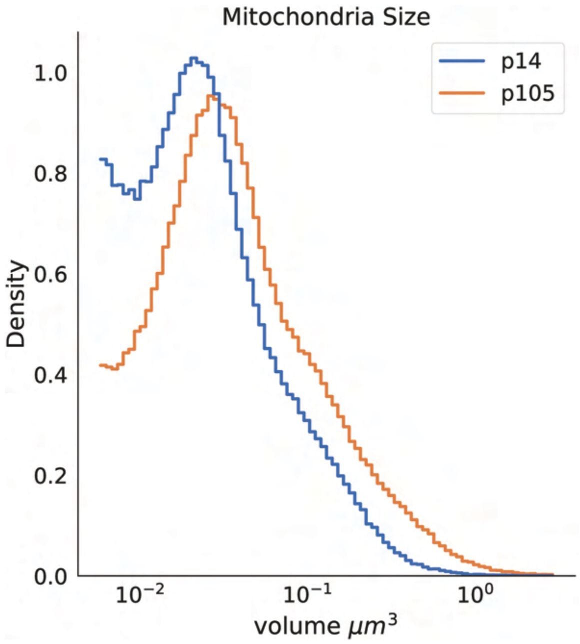

Supplementary Figure 9. UNET automatic segmentation and analysis of changes in mitochondria size in mouse p14 and p105, L4. a, 3D rendering of a representative subvolume depicting automatically segmented mitochondria from mouse p14 (left) and p105 (right). Each colored object represents an individual mitochondria. **b,** Density plot quantifying mitochondria size from automatic segmentation in mouse p14 (blue) and p105 (orange). Two-tailed Mann-Whitney U test. $p \sim 0$. Source data are provided as a Source Data file.

Supplementary Figure 10

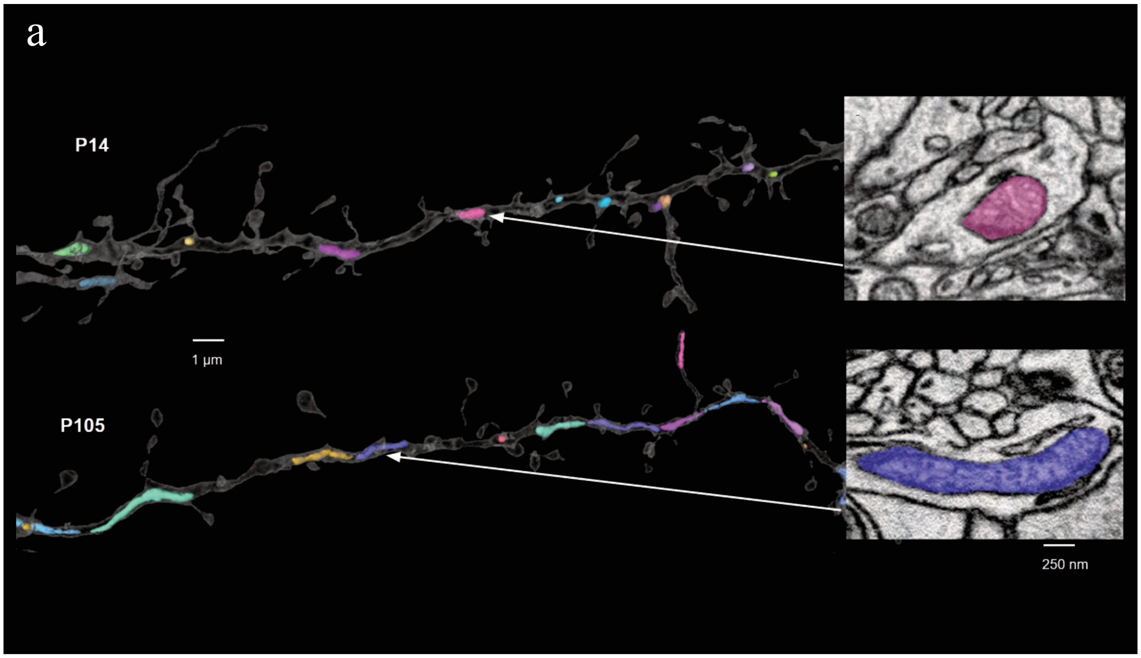

b

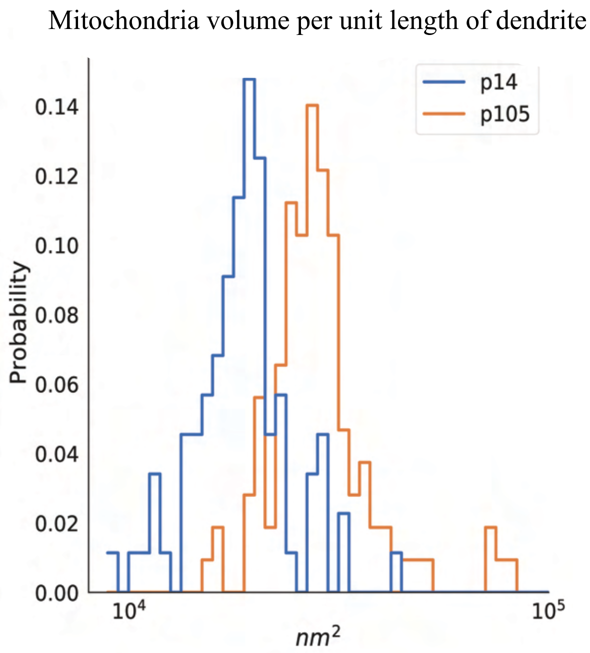

Supplementary Figure 10. Mitochondria density along dendrites in mouse p14 and p105, L4. a, Representative rendering from FFN segmentation of an excitatory dendrite from p14 (top) and p105 (bottom) containing UNET segmented mitochondria. *Right:* 2D EM image showing an example of one segmented mitochondria from each dendrite. **b,** Density plot quantifying mitochondria volume per unit length of dendrite. Scale bar: a, left= 1 μm; a, right=200 nm.Two-tailed Mann-Whitney U test. $p \sim$ 1e-20. Source data are provided as a Source Data file.

Supplementary Figure 11

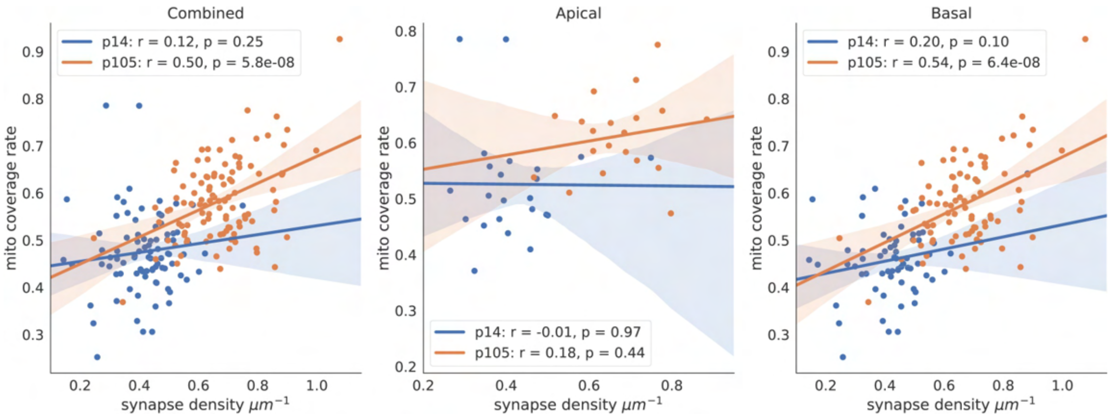

Supplementary Figure 11. Correlation between synapse density and mitochondria coverage. Scatter plots of x:synapse density and y:mitochondria coverage rate in p14 (blue) and p105 (orange) across *left*: apical and basal dendrites combined, *middle*: apical dendrites only, and *right*: basal dendrites only. Pearson correlation coefficient, combined: p14 r = 0.12, p = 0.25, p105 r = 0.5, p = 5.8e-8; apical: p14 r = -0.01, p = 0.97, p105 r = 0.18, p = 0.44; basal: p14 r = 0.2, p = 0.1, p105 r = 0.54, p = 6.4e-8. Two-tailed Mann-Whitney U test. Total automatically segmented and manually proofread synapse counts: mouse p14 = 6809, p105 = 13509. Source data are provided as a Source Data file.

Supplementary Figure 12

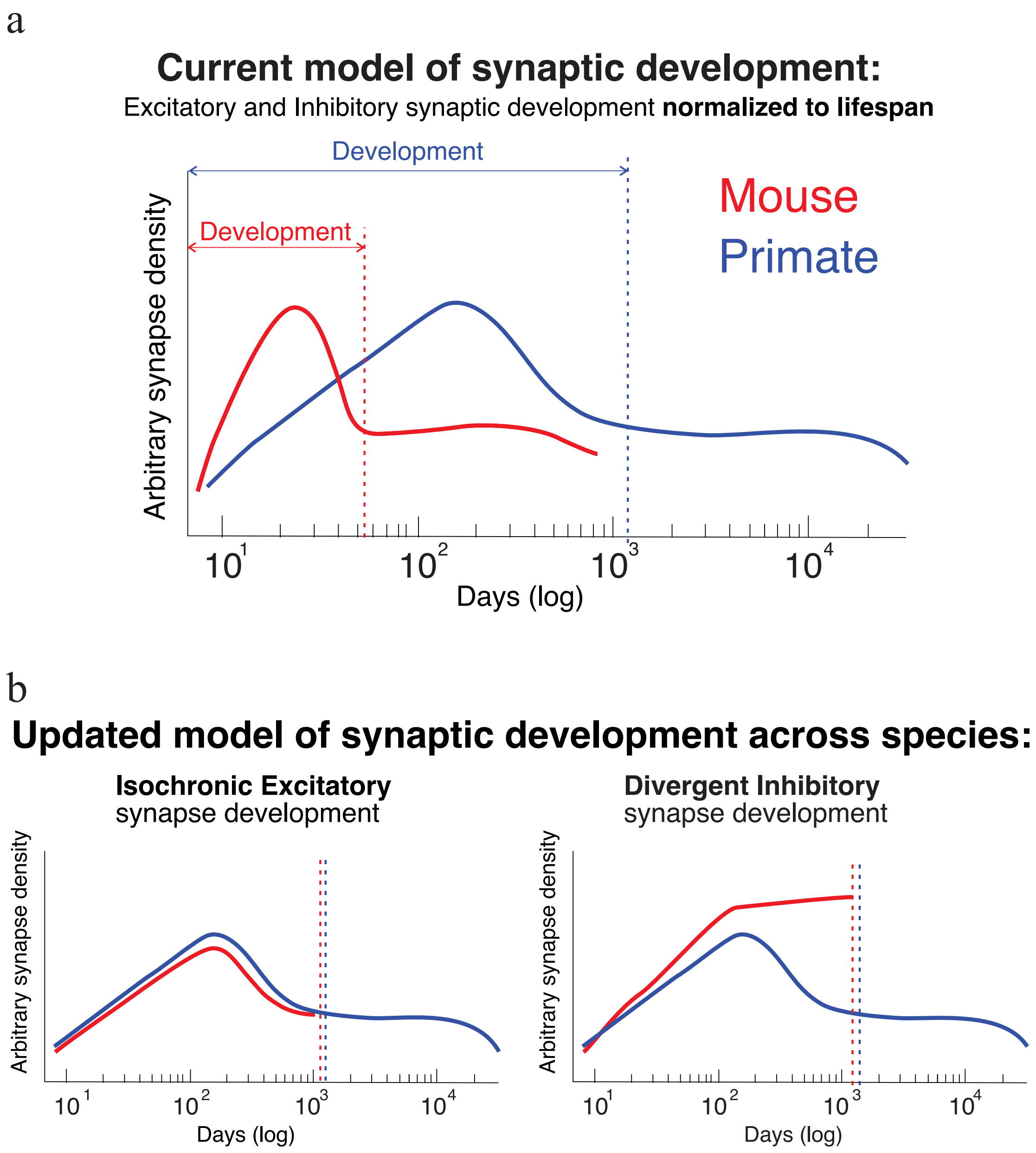

Supplementary Figure 12. An updated model of isochronic excitatory and inhibitory synapse development. a, Current models of synapse development assume that the cycle of synapse formation and pruning of both excitatory and inhibitory synapses occurs earlier in shorter lived, less cognitive species, like the mouse, and delayed in longer lived, species with more human like cognitive abilities (e.g., the macaque). **b,** Our new model of synaptic development in mouse and primate. Excitatory synapse formation and pruning is isochronic across species (i.e., a single synaptic clock) (left), whereas inhibitory somatic synapses in mice and primates show isochronic formation and primate inhibitory synapses are pruned at the same time as primate and mouse excitatory synapses. Mouse inhibitory synapses do not seem to prune and instead are continuously added throughout the life of the animal (right), revealing a qualitative difference in synaptic development across these species. Mouse = red lines, primate = blue lines

Supplementary Figure 13

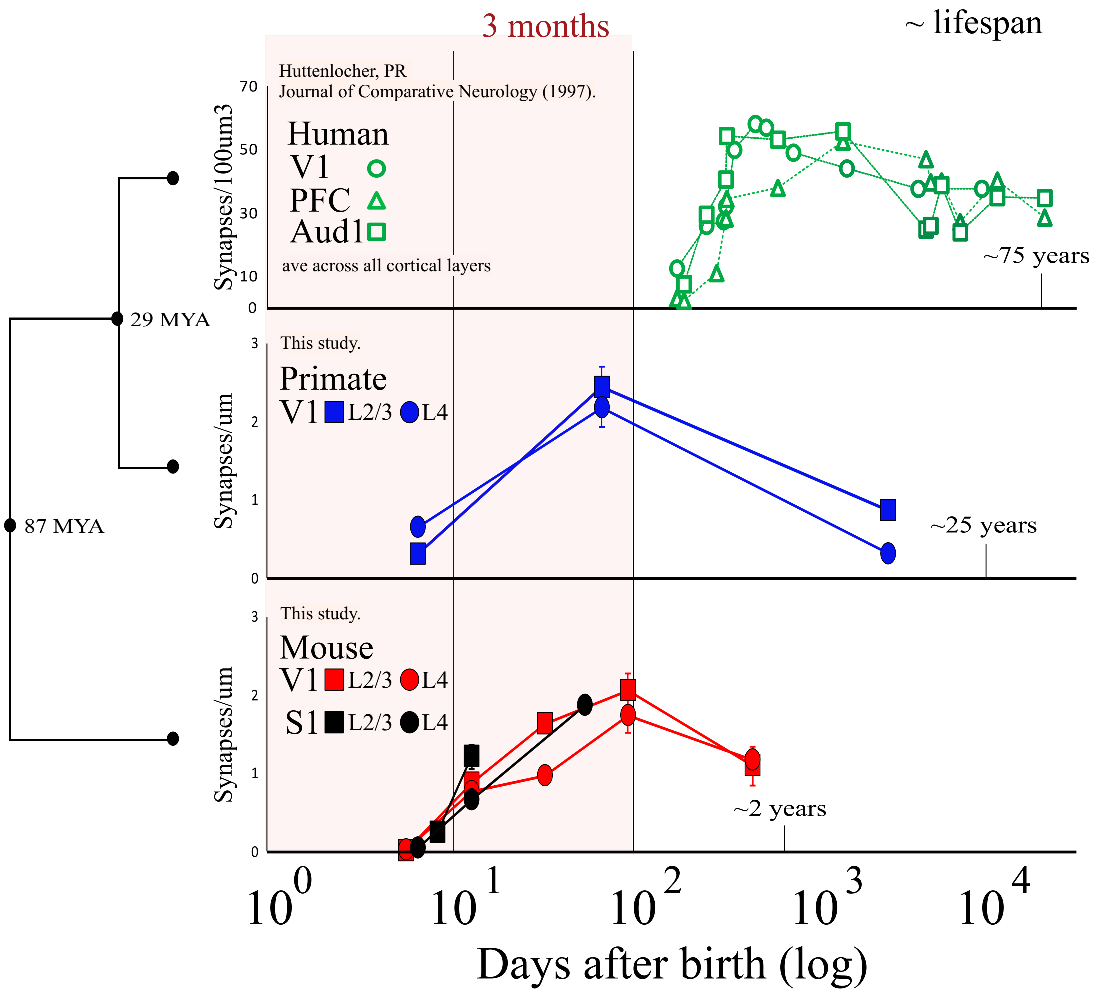

Supplementary Figure 13. Neoteny of excitatory synapse development in humans is a quantal evolutionary change. We scraped the existing literature on human synaptic development to compare with our results. Shown are scatter plots of x: days after birth (log) and y: synapse density counts for human (top), primate (middle), and mouse (bottom). Shown on the left is the number of millions of years ago (MYA) of the last common ancestor between human, primate, and mouse. For each dataset, species, cortical area, average lifespan and publication source is listed. V1 = primary visual cortex, PFC = prefrontal cortex, Aud1 = primary auditory cortex. Human synapse densities were taken directly from the original publication and re-plotted.

Supplementary Figure 14

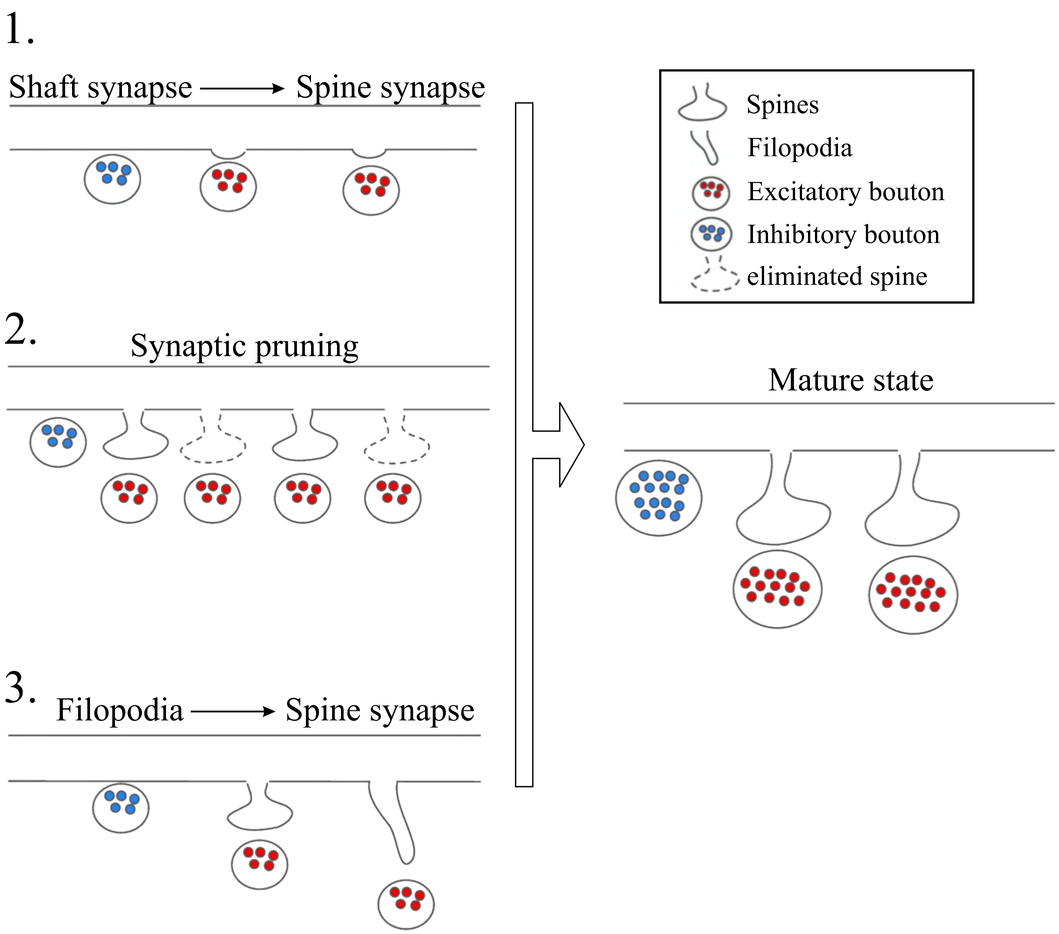

Supplementary Figure 14. Hypothetical models of excitatory spine synapse development. *Model 1*: in neonates, some synapses on dendritic shafts bud off the dendrite to form spines (red) while others remain as shaft synapses (blue). *Model 2*: A supernumerary number of spine synapses form in neonates and subsequently are pruned. *Model 3*: neonates form filopodia extensions off dendritic shafts that mature into spine synapses.

Supplementary Table 1

**Figure 1b**	p7	p75	p3000
Primate V1			
L2/3	0.31 ± 0.05,<br>n = 20, 10 µm dendrites	2.44 ± 0.27,<br>n = 19, 10 µm dendrites	0.86 ± 0.09,<br>n = 20, 10 µm dendrites
L4	0.65 ± 0.08,<br>n = 20, 10 µm dendrites	2.18 ± 0.24,<br>n = 15, 10 µm dendrites	0.32 ± 0.03,<br>n = 20, 10 µm dendrites

	p6	p14	p36	p87	p105	p523
Mouse V1						
L2/3	0.01 ± 0.007,<br>n = 20, 10 µm dendrites	0.87 ± 0.08,<br>n = 20, 10 µm dendrites	1.63 ± 0.11,<br>n = 20, 10 µm dendrites		2.06 ± 0.21,<br>n = 20, 10 µm dendrites	1.10 ± 0.25,<br>n = 20, 10 µm dendrites
L4	0.02 ± 0.01,<br>n = 19, 10 µm dendrites	0.77 ± 0.09,<br>n = 20, 10 µm dendrites		0.97 ± 0.07,<br>n = 20, 10 µm dendrites	1.74 ± 0.22,<br>n = 20, 10 µm dendrites	1.17 ± 0.13,<br>n = 20, 10 µm dendrites
Mouse S1	p9	p14	p60			
L2/3	0.25 ± 0.04,<br>n = 20, 10 µm dendrites	1.21 ± 0.2,<br>n = 20, 10 µm dendrites	not done			
L4	0.04 ± 0.01,<br>n = 20, 10 µm dendrites*	0.66 ± 0.1,<br>n = 20, 10 µm dendrites	1.87 ± 0.06,<br>n = 14, 10 µm dendrites			

*age is p7

**Figure 1c**	p7	p75	p3000
Primate V1			
L2/3	0.12 ± 0.03,<br>n = 20, 10 µm dendrites	0.18 ± 0.03,<br>n = 19, 10 µm dendrites	0.18 ± 0.04,<br>n = 20, 10 µm dendrites
L4	0.20 ± 0.03,<br>n = 20, 10 µm dendrites	0.23 ± 0.03,<br>n = 15, 10 µm dendrites	0.52 ± 0.08,<br>n = 20, 10 µm dendrites

	p6	p14	p36	p105	p523
Mouse V1					
L2/3	0.16 ± 0.03,<br>n = 20, 10 µm dendrites	0.1 ± 0.02,<br>n = 20, 10 µm dendrites	0.18 ± 0.03,<br>n = 20, 10 µm dendrites	0.20 ± 0.03,<br>n = 20, 10 µm dendrites	0.29 ± 0.04,<br>n = 20, 10 µm dendrites
L4	0.08 ± 0.02,<br>n = 19, 10 µm dendrites	0.08 ± 0.02,<br>n = 20, 10 µm dendrites	0.26 ± 0.02,<br>n = 20, 10 µm dendrites	0.26 ± 0.04,<br>n = 20, 10 µm dendrites	0.23 ± 0.06,<br>n = 20, 10 µm dendrites

Supplementary Table 1. Numerical averages, standard error of the mean (sem), and n number for quantification from Figure 1.

Supplementary Table 2

Figure 2-3

	excitatory axons			inhibitory axons		
	branches/ um	synapses/ um	axons /um^2	synapses/ soma	axons/ soma	bouton volume (um^3)
**Primate V1,L2/3**						
p7	0.16 ± 0.03, n = 50 axons	0.93 ± 0.1, n = 50 axons	5.9 ± 1.0, n = 3 FOVs, 6 um^2 average area/FOV	15.8 ± 2.1, n = 95 synapses across 6 soma	13.3 ± 2.7, n = 53 axons across 4 soma	0.08 ± 0.008, n = 50 boutons
p75	0.38 ± 0.04, n = 50 axons	1.0 ± 0.09, n = 50 axons	4.4 ± 0.7, n = 3 FOVs, 14 um^2 average area/FOV	50.3 ± 4.1, n = 302 synapses across 6 soma	22.4 ± 1.6, n = 112 axons across 5 soma	0.16 ± 0.02, n = 50 boutons
p3000	0.22 ± 0.03, n = 50 axons	0.46 ± 0.05, n = 50 axons	3.6 ± 0.6, n = 3 FOVs, 13 um^2 average area/FOV	16.1 ± 2.1, n = 129 synapses across 8 soma	12.5 ± 1.5, n = 75 axons across 6 soma	0.27 ± 0.03, n = 44 boutons
**Primate V1,L4**						
p7	not done	not done	not done	23.5 ± 2.7, n = 141 synapses across 6 soma	not done	not done
p75	not done	not done	not done	32.7 ± 4.9, n = 196 synapses across 6 soma	not done	not done
p3000	not done	not done	not done	14.2 ± 1.4, n = 85 synapses across 6 soma	not done	not done

	excitatory axons			inhibitory axons		
**Mouse V1,L2/3**	branches /um	synapses/ um	axons/ um^2	synapses/ soma *	axons/ soma	bouton volume (um^3)
p6	not done	not done	0.83 ± 0.05, n = 3 FOVs, 19.2 um^2 average area/FOV	1.2 ± 0.3, n = 7 synapses across 6 soma	not done	not done
p14	0.12 ± 0.02, n = 50 axons	0.71 ± 0.09, n = 50 axons	4.1 ± 0.14, n = 6 FOVs, 8.0 um^2 average area/FOV	25.8 ± 3.5, n = 155 synapses across 6 soma	17.3 ± 2.5, n = 69 axons across 4 soma	0.24 ± 0.03, n = 50 boutons
p105	0.22 ± 0.02, n = 74 axons	1.2 ± 0.07, n = 74 axons	4.4 ± 0.5, n = 6 FOVs, 9.1 um^2 average area/FOV	72.2 ± 5.1, n = 650 synapses across 9 soma	33 ± 2.5, n = 165 axons across 5 soma	0.26 ± 0.02, n = 84 boutons
p523	0.15 ± 0.02, n = 52 axons	0.86 ± 0.09, n = 52 axons	4.0 ± 0.8, n = 3 FOVs, 16 um^2 average area/FOV	79.2 ± 7.1, n = 475 synapses across 6 soma	39.3 ± 1.0, n = 157 axons across 4 soma	0.29 ± 0.03, n = 50 boutons
**Mouse V1,L4**						
p6	not done	not done	not done	2.2 ± 1.0, n = 13 synapses across 6 soma	not done	not done
p14	not done	not done	not done	11.3 ± 1.4, n = 45 synapses across 4 soma	not done	not done
p105	not done	not done	not done	42.9 ± 4.6 n = 45 synapses across 10 soma	not done	not done
p523	not done	not done	not done	53.8 ± 7.5, n = 164 synapses across 6 soma	not done	not done

*mouse p38 synapses/soma; L2/3: 66 ± 3.79, n = 396 synapses across 6 soma, L4: 38 ± 0.82, n = 228 synapses across 6 soma

Figure 4

	spine synapse density	filopodia density	synapses/soma
**Mouse V1, L4**			
p14	0.43± 0.11, n = 88 manually proofread dendrites	0.075± 0.038, n = 88 manually proofread dendrites	18.31± 12.15, n = 39
p105	0.69± 0.11, n = 107 manually proofread dendrites	0.005± 0.008, n = 107 manually proofread dendrites	42.85± 28.8, n = 42

Supplementary Table 2. Numerical averages, standard error of the mean (sem), and n number for quantification from Figure 2-3.

Supplementary Table 3

Dataset	dimensions (um)			animal gender	our lab generated (L) or public (P) data
Mouse V1,L2/3					
p6	x: 102.8	y: 94.5	z: 12.0	male	L
p14	x: 83.7	y: 75.3	z: 21.1	male	L
p36	https://www.microns-explorer.org/phase1			male	P
p105	x: 107.6	y: 99.7	z: 30.0	male	L
p523	x: 111.8	y: 95.6	z: 22.5	male	L
Mouse V1,L4					
p6	x: 136.6	y: 126.0	z: 18.8	male	L
p14	x: 60.0	y: 60.0	z: 24.0	male	L
p87	https://www.microns-explorer.org/cortical-mm3			male	P
p105	x: 126.1	y: 139.4	z: 18.7	male	L
p523	x: 95.2	y: 82.8	z: 22.6	male	L

Primate V1,L2/3					
p7	x: 121.4	y: 123.6	z: 16.0	male	L
p75	x: 134.8	y: 123.1	z: 38.0	male	L
p3000	x: 95.3	y: 90.9	z: 27.0	male	L
Primate V1,L4					
p7	x: 124.4	y: 128.7	z: 15.5	male	L
p75	x: 134.8	y: 124.9	z: 17.9	male	L
p3000	x: 120.5	y: 124.7	z: 14.6	male	L

Mouse S1,L2/3			
p9	https://pubmed.ncbi.nlm.nih.gov/33273061/	male	P
p14	https://pubmed.ncbi.nlm.nih.gov/33273061/	male	P
Mouse S1,L4			
p7	https://pubmed.ncbi.nlm.nih.gov/33273061/	male	P
p14	https://pubmed.ncbi.nlm.nih.gov/33273061/	male	P
p60	https://pubmed.ncbi.nlm.nih.gov/26232230/	male	P

Supplementary Table 3. Dimensions of each dataset (µm), gender of animal, and whether the dataset was originally generated for this study or pulled from public sources and reanalyzed. Public datasets contain url to original source.
