# Supplementary material for: Isochronic development of cortical synapses in primates and mice
Source: Nat Commun. 2023 Dec 4;14:8018. doi: 10.1038/s41467-023-43088-3 (PMC10695974; doi:10.1038/s41467-023-43088-3)
Supplement: Supplementary file 3 — Reporting Summary [file 41467_2023_43088_MOESM3_ESM.pdf]

nature portfolio

Corresponding author(s): Narayanan Kasthuri

Last updated by author(s): Oct 11, 2023

Reporting Summary

Nature Portfolio wishes to improve the reproducibility of the work that we publish. This form provides structure for consistency and transparency in reporting. For further information on Nature Portfolio policies, see our Editorial Policies and the Editorial Policy Checklist.

Statistics

For all statistical analyses, confirm that the following items are present in the figure legend, table legend, main text, or Methods section.

n/a	Confirmed	
☐	☒	The exact sample size (n) for each experimental group/condition, given as a discrete number and unit of measurement
☐	☒	A statement on whether measurements were taken from distinct samples or whether the same sample was measured repeatedly
☐	☒	The statistical test(s) used AND whether they are one- or two-sided<br>*Only common tests should be described solely by name; describe more complex techniques in the Methods section.*
☐	☒	A description of all covariates tested
☐	☒	A description of any assumptions or corrections, such as tests of normality and adjustment for multiple comparisons
☐	☒	A full description of the statistical parameters including central tendency (e.g. means) or other basic estimates (e.g. regression coefficient) AND variation (e.g. standard deviation) or associated estimates of uncertainty (e.g. confidence intervals)
☐	☒	For null hypothesis testing, the test statistic (e.g. F, t, r) with confidence intervals, effect sizes, degrees of freedom and P value noted<br>*Give P values as exact values whenever suitable.*
☐	☒	For Bayesian analysis, information on the choice of priors and Markov chain Monte Carlo settings
☐	☒	For hierarchical and complex designs, identification of the appropriate level for tests and full reporting of outcomes
☐	☒	Estimates of effect sizes (e.g. Cohen's d, Pearson's r), indicating how they were calculated

Our web collection on statistics for biologists contains articles on many of the points above.

Software and code

Policy information about availability of computer code

Data collection	We used commercial SEM (Zeiss) software to acquire images.
Data analysis	We used the ImageJ(FUIJI) plugin TrakEM2 and the open source software AlignTK to create 3d EM volumes. We used the program knossos to generate most of the reconstructions. We used custom MATLAB code for most data analyses and we used custom python code to measure neuronal distances etc. All code has been deposited on GitHub.

For manuscripts utilizing custom algorithms or software that are central to the research but not yet described in published literature, software must be made available to editors and reviewers. We strongly encourage code deposition in a community repository (e.g. GitHub). See the Nature Portfolio guidelines for submitting code & software for further information.

Data

Policy information about availability of data

All manuscripts must include a data availability statement. This statement should provide the following information, where applicable:

- Accession codes, unique identifiers, or web links for publicly available datasets
- A description of any restrictions on data availability
- For clinical datasets or third party data, please ensure that the statement adheres to our policy

All original EM data for V1 are freely available online at: https://bossdb.org/project/wildenberg2023 or DOI:10.60533/boss-2023-0s41. Mouse S1 datasets that we analyzed for changes in excitatory synapses are available at their original publication31 which can be found at: https://pubmed.ncbi.nlm.nih.gov/33273061/.

Mouse p60, S1 excitatory synapse measurements were manually analyzed using the publicly available neuroglancer file from104 which can be found here: https://github.com/google/neuroglancer. Mouse p36 datasets are publicly available here for L2/3: https://www.microns-explorer.org/phase1 and here for, p87, L4: https://www.microns-explorer.org/cortical-mm3. Automatic segmentation of neurons, synapses and mitochondria of mouse V1, L4 p14 and p105 are available as a WebKnossos format available for further public proofreading/error checking. Source data are provided with this paper: for Figures 1-3, source data can be found in the source data excel file. All other source data (i.e., Figure 4 and Supplementary Figures 7-11) can be found here: https://bossdb.org/project/wildenberg2023.

Research involving human participants, their data, or biological material

Policy information about studies with human participants or human data. See also policy information about sex, gender (identity/presentation), and sexual orientation and race, ethnicity and racism.

Reporting on sex and gender	N/A
Reporting on race, ethnicity, or other socially relevant groupings	N/A
Population characteristics	N/A
Recruitment	N/A
Ethics oversight	N/A

Note that full information on the approval of the study protocol must also be provided in the manuscript.

Field-specific reporting

Please select the one below that is the best fit for your research. If you are not sure, read the appropriate sections before making your selection.

[x] Life sciences [ ] Behavioural & social sciences [ ] Ecological, evolutionary & environmental sciences

For a reference copy of the document with all sections, see nature.com/documents/nr-reporting-summary-flat.pdf

Life sciences study design

All studies must disclose on these points even when the disclosure is negative.

Sample size	Sample sizes were not predetermined. We annotated numerous neuronal features to test our hypothesis of neontenous development of synapses. For each feature being manually annotated (e.g., spine synapses), we randomly chose these features (e.g., spinous dendrites) and had more than one person annotating the data. The number of objects to annotate was based on what was feasible with manual annotations at which point we performed a statistical test using a two-tailed Mann Whitney U test. For automatic annotations, 1,000's of features were unbiasedly segmented using machine learning algorithms.
Data exclusions	no data were excluded from our analyses
Replication	at the request of one reviewer, we replicated results from our p6 mouse synapse density results by preparing and imaging EM sections from 5 separate p6 mice. For all other data, we performed "replications" of the data annotation by having multiple annotators randomly check ~30% of another persons annotations. For automatic segmentations, we only report data that was manually proofread by two people.
Randomization	samples were organized by age. Each age had a n=1 dataset.
Blinding	proofreading of annotations (see Replication) was blind: annotators prooreading other's work were blind to the dataset specifics.

Reporting for specific materials, systems and methods

We require information from authors about some types of materials, experimental systems and methods used in many studies. Here, indicate whether each material, system or method listed is relevant to your study. If you are not sure if a list item applies to your research, read the appropriate section before selecting a response.

Materials & experimental systems

n/a	Involved in the study
☒	☐ Antibodies
☒	☐ Eukaryotic cell lines
☒	☐ Palaeontology and archaeology
☐	☒ Animals and other organisms
☒	☐ Clinical data
☒	☐ Dual use research of concern
☒	☐ Plants

Methods

n/a	Involved in the study
☒	☐ ChIP-seq
☒	☐ Flow cytometry
☒	☐ MRI-based neuroimaging

Animals and other research organisms

Policy information about studies involving animals; ARRIVE guidelines recommended for reporting animal research, and Sex and Gender in Research

Laboratory animals	C57BL/6 mice and Rhesus macaque post mortem tissue was used for this study. All ages noted in manuscript.
Wild animals	No wild animals were used
Reporting on sex	All animals used in the study were male
Field-collected samples	The study did not involve samples collected from the field.
Ethics oversight	All experimental aspects conducted complies with The University of Chicago Institutional Animal Care and Use Committee (IACUC), Animal Resource Center and Office of Research Safety

Note that full information on the approval of the study protocol must also be provided in the manuscript.

Plants

Seed stocks	No plants were used in this study.
Novel plant genotypes	N/A
Authentication	N/A
